# Extremely asynchronic delivery of second and third fetuses in a triple pregnancy

**DOI:** 10.5935/1518-0557.20140007

**Published:** 2014

**Authors:** Cecilia Fabres, Alfredo M. Germain, Lorena Quiroz, Javier A. Crosby

**Affiliations:** 1 Reproductive Medicine Unit, Department of Obstetrics & Gynecology, Clinica las Condes, Santiago, Chile; 2 Maternal Fetal Medicine Unit, Department of Obstetrics & Gynecology, Clinica las Condes, Santiago, Chile

**Keywords:** Music therapy, infertility, assisted reproduction, stress

## Abstract

Report of clinical treatment of a patient with a triple pregnancy after ICSI, who had the abortion of the first fetus at 16 weeks of gestation and the “asynchronic delivery” of the other two, at 28 weeks. A reproductive inflammatory process previously diagnosed in the couple could have been related with the premature rupture of membranes (PROM) occurred at 15.5 weeks of pregnancy. The clinical interventions described, made possible the delayed delivery and the survival of the other two triplets. This case shows us the importance to transfer no more than two embryos during ART, to avoid the catastrophic consequences of a triple pregnancy.

## INTRODUCTION

Multiple pregnancies present maternal and fetal complications such as fetal loss before 24 weeks, early preterm delivery before 32 weeks, pre-eclampsia and gestational diabetes. Perinatal mortality rate for twin pregnancies is between 47-120/1000 newborns and for triplets, 92-203/1000 newborns (Lipitz *et al*., 1989). The incidence of high order multiple pregnancies correlates directly with the number of embryos transferred after IVF (Crowther, 1999).

The probability of a premature baby to survive is directly correlated with the number of days that remains in uterus during the second trimester of pregnancy (Fanaroff *et al*. 1995). It has been demonstrated that before 30 weeks of pregnancy, even a delay of two or more days in labor, may improve the survival rate from 24 to 56% (Zhang *et al*, 2004).

In a multiple pregnancy, when one of the fetuses is aborted, some strategies have been implemented to allow the others stay in uterus for longer time, delaying labor (Yuce *et al*., 2010).

## CASE REPORT

The case corresponded to an infertile couple referred to our center due to primary infertility of 5.5 years. They lived in a town far away from the capital city. He was 35 years old, had a varicocele, normal sperm count but with astheno-terato-zoospermia and increased number of leucocytes. He had been treated with antibiotics and anti-inflammatories. She was 34 years old, overweight (BMI = 29), normal clinical background, no abdominal surgeries, regular menses every 35 days and a normal hysterosalpingogram. After a complete study, their final diagnosis was: polycystic ovary like syndrome associated to insulin resistance, uterine subserous fibroid (44 x 44 mm), non-specific chronic endometritis (endometrial biopsy) and chronic epididymitis (positive semen cultures for Chlamydia Thrachomatis). The prevalence of this infection in our population, is very low (< 3%). They had an inflammatory reproductive process (IRP)

The couple received treatment with anti-inflammatories and antibiotics of wide spectrum infection during approximately one month. He also received antioxidants for longer time to diminish oxidative stress on sperm, caused by his chronic infection. Semen cultures became negative. She started treatment with Metformin (Glafornil XR^®^, Merck Serono), hypocaloric diet and exercise.

A year later, they came for an ICSI, initiating controlled ovarian stimulation with a step down protocol (225 IU of rFSH (Gonal-f^®^, Merck Serono) as initial dose and 0.5 mg of leuprolide acetate (Lupron^®^, Abbott) as GnRH agonist). The routine serologic screening performed before the ICSI was negative (HIV, RPR, Cytomegalovirus, Rubella, HBV, HCV). Ovum pick up under transvaginal ultrasound vision was performed 36 hours after hCG administration (250µg Ovidrel^®^, Merck Serono), recovering 14 matured oocytes that were injected by ICSI, obtaining eleven zygotes. Three embryos were transferred on the 3rd day of development and two blastocysts were vitrified on the 5th day. The lutheal support was achieved with Fertiring^®^ (Silesia) plus daily 200 mg of intravaginal progesterone (Progendo^®^, Recalcine). She became pregnant and at 6 weeks, the ultrasound back home showed a tri chorionic triple pregnancy. They were exhorted to come for a control, to evaluate the length of the cervix or the presence of a cervical infection (due their past history of IRP), and the possibility of a preventive cerclage, but they didnt. We didnt have information about medical evaluations during the first trimester.

At 15.5 weeks of pregnancy she had a premature rupture of membranes (PROM) of the first gestational sac and her OB & Gyn doctor, admitted her in the hospital with spontaneous evolution. Three days later, being under antibiotic treatment, abortion had not occurred and they decided to continue treatment in our center. She was transported in a plane to Santiago (capital city).

At admission, abdominal ultrasound confirmed a severe oligohydramnios of the first triplet, who was still alive. The other two sacs had normal amount of amniotic fluid, fetuses were growing according to gestational age and had normal cardiac frequency.

All placentas were separated and adequate for gestational age. Cervical cultures for aerobic and anaerobic bacteria were negative, but a vaginal fluid PCR test, was positive for Enterococcus faecium. Antibiotic scheme was maintained. Full blood test showed leucocyte count of 10.690/mL with 2% bacilliforms. CRP = 6mg/Lt and normal vital signs, without fever or tachycardia.

Spontaneous evolution was decided, maintaining frequent clinical and laboratory controls, which were all in normal range. The death and the inevitable abortion of the first triplet occurred on the fourth day of evolution.

The umbilical cord was ligated, close to the placenta and cut in the usual way.

The placenta was left inside the uterus and a Shirodkar cerclage was performed using mersilene string (1 x Cervix Set, B Braun, Aesculap AG). After the cerclage abdominal ultrasound showed a cervical length of 45mm and tocolysis with Atosiban (Tractocile^®^, Ferring) was started.

IV antibiotic scheme was maintained for other seven days, together with permanent vaginal metronidazol 500 mg suppositories (according to Clinica las Condes´ Protocol of Intra-hospital Infections).

The anatomo-pathological study of the aborted fetus concluded: male fetus adequate for gestational age, with signs of intrauterine hypoxia (cyanosis, edema and multiple visceral micro hemorrhages), without signs of fetal infection. The patient remained with complete bed rest and frequent clinical and laboratory controls every 24 and 48 hours respectively, that were always without signs of chorioamnionitis. She was permanently using anti embolic stalkins and subcutaneous low molecular weight heparin.

At 19.5 weeks, after 24 days of hospitalization, the patient was discharged maintaining regular controls in the clinic every 2 weeks.

During the 24th week of pregnancy, gestational diabetes was detected (GTT 75 g, 83/174 mg/Dl) even though she had been under Metformin treatment before and during her pregnancy. On week 26, an abdominal ultrasound detected polyhydramnios in gestational sac one (former gestational sac 2), the fetus was growing in percentile 50-75, gestational sac two (former gestational sac 3), had normal amount of amniotic fluid, and the fetus was growing in percentile 75. The uterine cervix length was 35 mm.

At 28.4 weeks of gestation the patient was readmitted in the hospital with a PROM of gestational sac 1.

Ultrasound evidenced anhydramnios in this sac and normal amniotic fluid in gestational sac 2. Pulmonary maturation was induced with corticosteroids as usual.

Infectious screening of the uterine cervix showed persistence of Enterococcus. IV antibiotic treatment (Teicoplanin and Ceftriaxona) was initiated. Three days later, she started labor work and a cesarean section was performed 82 days after the abortion of the first triplet. First twin: female, cephalic presentation, 985 g, 34 cm, APGAR 8/9. Second twin: male, breech presentation, 1110 g, 36 cm, APGAR 8/8.

Histological study of the placentas informed that both had normal characteristics for gestational age. The third placenta left inside the uterus after the abortion, weighted 53 g and showed extended infarct areas partially organized. The female newborn presented respiratory distress with hyaline membrane; she received two doses of surfactant and a transfusion of one unit of red blood cells for a severe anemia of the premature, being extubated 24 hours after birth.

Other diagnoses were: hyper bilirubinemia and apneic syndrome of the premature. The neurological, cardiac, renal, ophthalmological, bone and metabolic evaluations were all in normal range. She was discharged from the hospital at 60 days of life weighting 2165 g.

The male newborn also presented respiratory distress with hyaline membrane; he received two doses of surfactant with good clinical evolution.

Extubation was decided after 12 hours, presenting several episodes of apnea and cardio respiratory arrest, he died on the third cardiac arrest 24 hours after birth. Bronco pulmonary hemorrhage was diagnosed evidenced by the presence of fresh blood coming out the endo tracheal tube.

## DISCUSSION

The review of the literature about delayed delivery in triple pregnancies after early abortion of one fetus shows poor results.

Several conditions must be present to apply the strategy of a delayed delivery in a multiple pregnancy when the first triplet aborts due to PROM (Arabin & Van Eyck, 2009): triplets should have separate amniotic sacs, the remained fetuses should have intact amniotic membranes, absence of fetal distress, abruptio placentae, amniotic infection and/or maternal indication to proceed with labor (Kalchbrenner *et al*., 1998; Van der Streaten *et al*. 2001), all these conditions were present in this case. The patient had no other risk factor for PROM.

The recommendation is that after the abortion, the umbilical cord be cut as close to the placenta as possible, with absorbable suture, living the placenta inside the uterus (Van der Streaten *et al*. 2001).

The use of a cerclage is controversial (Arabin & Van Eyck, 2009), it should be indicated mainly when the cervix remains dilated after the abortion, as was this case. It may give stability to the cervix and is associated with longer interval between the abortion and delivery of the remained fetuses, however other authors consider that may increase the risk of intra amniotic infections (Arabin & Van Eyck, 2009).

Different schemes of intravenous wide spectrum antibiotics are recommended during short periods, changing the scheme according to the pathogens found in vaginal cultures. Tocolysis is also recommended as well as corticosteroids to induce pulmonary maturation after 24 weeks of gestation, when labor approaches (Zhang *et al*.,2003). Careful monitoring of the patient to detect early amniotic infection, preterm labor, fetal distress or abruptio placentae is recommended. Vital signs and temperature must be controlled every 6-8 hours. Leukocyte count and CRP values every 24 to 48 hours.

Vaginal cultures should be done weekly. Also evaluation of fetal wellness, with ultrasonic biometry, fetal Doppler, amniotic fluid amount and cardio tocography are recommended weekly (Arabin & Van Eyck, 2009; Zhang *et al*.,2003). This was a case of “asynchronic delivery” in a triple pregnancy, the first one reported with the abortion of the first triplet as early as 16 weeks and the fourth one, with a difference of 82 days between the abortion of the first triplet and the delivery of the other two.

This case points out the importance to avoid high order multiple pregnancies and their catastrophic consequences when more than two embryos are transferred.

It has been proved that by transferring more embryos, pregnancy rate do not increase, but there is an increment of multiple pregnancies (Zegers-Hochschild *et al*., 2013). Today, the baby girl is 24 month old; remained without sequelae and has a completely normal physical and psycho/ motor development for her corrected age. On September 2013, after the transference of two vitrified blastocysts, the patient delivered a healthy baby boy (3245 g; 49 cm; APGAR 9-9).
